# MIxS-SA: a MIxS extension defining the minimum information standard for sequence data from symbiont-associated micro-organisms

**DOI:** 10.1038/s43705-022-00092-w

**Published:** 2022-02-01

**Authors:** Fátima Jorge, Jaelle C. Brealey, Paul J. Brindley, Marie Buysse, Cinzia Cantacessi, Olivier Duron, Raina Fichorova, Connor R. Fitzpatrick, Megan Hahn, Christopher Hunter, Vincent Hervé, Laura J. Knoll, Kevin D. Kohl, Marco Lalle, Julius Lukeš, Joaquín Martínez Martínez, Susan L. Perkins, Robert Poulin, Karyna Rosario, Adam C. Schneider, Lynn M. Schriml, Luke R. Thompson, Ramona L. Walls, Nolwenn M. Dheilly

**Affiliations:** 1 Department of Zoology, University of Otago, Dunedin, New Zealand; 2 Department of Natural History, NTNU University Museum, Norwegian University of Science and Technology, Trondheim, Norway; 3 Department of Microbiology, Immunology and Tropical Medicine, School of Medicine & Health Sciences, George Washington University, Washington, D.C 20037 USA; 4 MIVEGEC (Maladies Infectieuses et Vecteurs: Ecologie, Génétique, Evolution et Contrôle), Centre National de la Recherche Scientifique (CNRS)—Institut pour la Recherche et le Développement (IRD) - Université de Montpellier (UM), Montpellier, France; 5 CREES (Centre de Recherche en Écologie et Évolution de la Santé), Montpellier, France; 6 Department of Veterinary Medicine, University of Cambridge, Cambridge, CB3 0ES UK; 7 Department of Obstetrics, Gynecology and Reproductive Biology, Brigham and Women’s Hospital, Harvard Medical School, Boston, MA USA; 8 Biology Department, University of North Carolina at Chapel Hill, Chapel Hill, NC USA; 9 New York City Department of Health and Mental Hygiene, Long Island City, NY USA; 10 GigaDB Director, GigaScience Press, BGI-Hong Kong, Hong Kong, China; 11 Institut de Recherche sur la Biologie de l’Insecte, UMR 7261, CNRS—Université de Tours, Avenue Monge, Parc Grandmont, 37200 Tours, France; 12 Department of Medical Microbiology and Immunology, University of Wisconsin-Madison, Madison, WI USA; 13 Department of Biological Sciences, University of Pittsburgh, Pittsburgh, PA 15260 USA; 14 Department of Infectious Diseases, Foodborne and Neglected Parasitic Diseases; European Union Reference Laboratory for Parasite, Istituto Superiore di Sanità, Rome, Italy; 15 Institute of Parasitology, Biology Centre, Czech Academy of Sciences, and Faculty of Sciences, University of South Bohemia, České Budějovice (Budweis), České Budějovice, Czech Republic; 16 Bigelow Laboratory for Ocean Sciences, East Boothbay, ME USA; 17 Department of Biology, The City College of New York, New York, NY 10031 USA; 18 College of Marine Science, University of South Florida, Saint Petersburg, FL USA; 19 Biology and Health Sciences Department, Hendrix College, Conway, AR USA; 20 Institute for Genome Sciences, Department of Epidemiology and Public Health, University of Maryland School of Medicine, Baltimore, MD 21201 USA; 21 Northern Gulf Institute, Mississippi State University, Mississippi State, MS 39762 USA; 22 Ocean Chemistry and Ecosystems Division, Atlantic Oceanographic and Meteorological Laboratory, National Oceanic and Atmospheric Administration, Miami, FL 33149 USA; 23 Data Collaboration Center, Critical Path Institute, Tucson, AZ USA; 24 ANSES, Agence Nationale de Sécurité Sanitaire de l’Alimentation, de l’Environnement et du Travail - Laboratoire de Ploufragan-Plouzané, Unité Génétique Virale de Biosécurité, Ploufragan, France; 25 Anses, INRAE, Ecole Nationale Vétérinaire d’Alfort, UMR VIROLOGIE, Laboratoire de Santé Animale, 94700 Maisons-Alfort, France

**Keywords:** Microbiome, Genomics

## Abstract

The symbiont-associated (SA) environmental package is a new extension to the minimum information about any (x) sequence (MIxS) standards, established by the Parasite Microbiome Project (PMP) consortium, in collaboration with the Genomics Standard Consortium. The SA was built upon the host-associated MIxS standard, but reflects the nestedness of symbiont-associated microbiota within and across host-symbiont-microbe interactions. This package is designed to facilitate the collection and reporting of a broad range of metadata information that apply to symbionts such as life history traits, association with one or multiple host organisms, or the nature of host-symbiont interactions along the mutualism-parasitism continuum. To better reflect the inherent nestedness of all biological systems, we present a novel feature that allows users to co-localize samples, to nest a package within another package, and to identify replicates. Adoption of the MIxS-SA and of the new terms will facilitate reports of complex sampling design from a myriad of environments.

## Introduction

Interspecific interactions are ubiquitous across the Tree of Life. With the realization that eukaryotic organisms can harbor rich microbial communities, came also the view that these smaller partners may in fact play important roles in mediating host-symbiont associations, thus adding a further layer to this complex set of nested interactions, *i.e*. host-symbiont-microbe [1–8]. As the number of studies exploring the microorganisms associated with symbiotic organisms increases, likewise does the need for compliant standardized metadata that provides contextual information associated with each study and sample. Standardized metadata allows for the integration of data across organisms, resources, and within data repositories. Here, we present the symbiont-associated (SA) environmental package as a new extension to the minimum information about any (x) sequence (MIxS) standards [9], which will be included in MIxS version 6. Whilst the MIxS-SA expands upon the MIxS host-associated environmental package [9], it reflects the need for a new standard that takes into account the distinct life history traits of symbionts, their association with one or multiple host organisms, the complex nature of host-symbiont interactions along the mutualism-parasitism continuum, and the nestedness of symbiont-associated microbiota. We also propose adding the term ‘relationship to other packages’ to all environmental packages across the domains of life, to allow for integrated analysis of symbiont and host microbiota by linking metadata elements across environmental packages. This will allow users to nest a package within another package, and to identify replicates. This added feature is pivotal for the study of the microbiome of symbionts that are themselves nested within a host, reflects the inherent nestedness of all ecosystems and will facilitate reports of complex sampling design from a myriad of environments.

Collecting relevant metadata (data describing data) is now widely recognized as critical to contextualize samples and increase their reusability and reproducibility [9–11]. The Genomic Standards Consortium (GSC, https://gensc.org) has developed and maintains a suite of minimal information metadata standards for describing sequence metadata (checklists) for genome (MIGS), metagenome (MIMS), marker gene sequences (MIMARKS), simple amplified genome (MISAG), metagenome-assembled genome (MIMAG), virus genomes (MIUViG) and environmental packages for describing habitat-specific contextual data of the sampling environment [9, 10, 12, 13], collectively referred to as the Minimum Information about any (x) Sequence (MIxS) standard (ref. [9], https://gensc.org/mixs/).

The MIxS standards are used broadly across the microbiome research communities. These standards have been integrated into large scale microbiome projects (e.g. Human Microbiome Project, https://www.hmpdacc.org/), Earth Microbiome Project (https://earthmicrobiome.org/), Microbiology of the Built Environment (MoBE, https://www.microbe.net), microbiome bioinformatics platforms (e.g., QIIME, Qiita, mothur, JGI GOLD, MG-RAST, EBI, NCBI) and are now required upon manuscript submission. A primary advantage of the MIxS standards is the collation of large aggregates of associated metadata that can be harnessed to uncover, and eventually comprehend, patterns of microbial diversity and ecology.

The MIxS-SA package was initially drafted during the 1st Parasite Microbiome project workshop that involved the contribution of members of the GSC in addition to microbial ecologists, parasitologists, pathologists and marine biologists [14]. Participants rapidly identified the need to incorporate information on the nestedness of symbiont-associated systems, and the absence within the MIxS host-associated package of descriptors of complex life histories of mutualistic and parasitic symbionts. Until now, researchers have either omitted this information or added research-specific symbiont-associated annotations, limiting significantly the potential to compare, combine and/or reuse data from different systems and studies. Whereas the MIxS-SA package was initially designed for the study of parasite-microbes interaction, the scope of the package was expanded to include non-parasitic symbionts. This addition is a necessary expansion due to the context-dependent nature of symbiotic interactions and the ability of a given symbiont to interact differently with different organisms. Notably, the resulting MIxS-SA package reduces the need to develop additional highly similar packages for different types of symbionts.

Symbiotic associations are generally classified as mutualistic (mutually beneficial association), commensal (beneficial association to one of the partners, but not harmful to the other), or parasitic (detrimental association to one of the partners) [15]. In the context of the symbiont-associated package, the term symbiont applies to macro and microorganisms that can establish a physical interaction with at least one other organism at some stage of their life cycle regardless of the nature and dependence of the interaction. As such, this definition also covers symbiotic organisms that establish facultative and accidental associations (e.g., dead-end hosts), not requiring evolutionary processes to explain their association, but excludes free-living organisms that establish a symbiotic relationship with another free-living organism (e.g., flowers and bees). The MIxS-SA package presented herein has gone through an open and iterative review process engaging the GSC community and experts studying symbiotic organisms across various symbiont and host taxa.

Here, we present the selected list of metadata descriptors for symbiont-associated microbiota studies, including a subset of mandatory (M) terms that underpin metadata compliance (Table 1; Supplementary Information SI-1 contains all MIxS-SA items). In order to allow comparative studies of the microbiota of, sometimes closely related, free-living and symbiotic organisms, the MIxS-SA includes terms already found in the MIxS host-associated package. Thus, in MixS-SA, the term “host” (when used alone) refers to the host of the biological sample which is the symbiotic organism. New terms were created to characterise the “host of the symbiotic host”. We provide symbiont-associated package specific “Expected values” and “Examples”. Changes to the package (addition of terms, modification etc.) can be proposed by the community by creating a ticket on the MIxS GitHub page (https://github.com/GenomicsStandardsConsortium/mixs).

**Table 1:** MIxS symbiont-associated environmental package representative terms, along with requirement status, description and MIXS IDs.

MIxS Package	Metadata category	Package item	Req	Definition	MIXS ID
Symbiont-associated	Symbiont specific descriptors	host dependence	M	Type of host dependence for the symbiotic host organism to its host.	0001315
		type of symbiosis	C	Type of biological interaction established between the symbiotic host organism being sampled and its respective host.	0001307
		symbiotic host organism life cycle type	M	Type of life cycle of the symbiotic host species (the thing being sampled). Simple life cycles occur within a single host, complex ones within multiple different hosts over the course of their normal life cycle.	0001300
		host life stage	M	Description of life stage of host.	0000251
		mode of transmission	C	The process through which the symbiotic host organism entered the host from which it was sampled.	0001312
		route of transmission	O	Description of path taken by the symbiotic host organism being sampled in order to establish a symbiotic relationship with the host (with which it was observed at the time of sampling) via a mode of transmission (specified in mode_transmission).	0001316
		host number individual	O	Number of symbiotic host individuals pooled at the time of collection.	0001305
	Symbiont – host relationship descriptors	observed host symbionts	O	The taxonomic name of the organism(s) found living in mutualistic, commensalistic, or parasitic symbiosis with the specific host. For cases when the specific host of the sample is a symbiont this field should refer to other organisms it is associated with: e.g.: hyperparasite species X (parasite of the parasite).	0001309
		host specificity	C	Level of specificity of symbiont-host interaction: e.g. generalist (symbiont able to establish associations with distantly related hosts) or species-specific.	0001308
		host of the symbiont role	C	Role of the host in the life cycle of the symbiotic organism.	0001303
		host cellular location	C	The localization of the symbiotic host organism within the host from which it was sampled: e.g., intracellular if the symbiotic host organism is localized within the cells or extracellular if the symbiotic host organism is localized outside of cells.	0001313
		duration of association with the host	O	Time spent in host of the symbiotic organism at the time of sampling; relevant scale depends on symbiotic organism and study.	0001299
		observed coinfecting organisms in host of host	O	The taxonomic name of any coinfecting organism observed in a symbiotic relationship with the host of the sampled host organism. e.g. where a sample collected from a host trematode species (A) which was collected from a host_of_host fish (B) that was also infected with a nematode (C), the value here would be (C) the nematode {species name} or {common name}. Multiple coinfecting species may be added in a comma-separated list. For listing symbiotic organisms associated with the host (A) use the term Observed host symbiont.	0001310
	Host of the symbiont descriptors	host of the symbiotic host common name	O	Common name of the host of the symbiotic host organism.	0001324
		host of the symbiotic host local environmental context	O	For a symbiotic host organism the local anatomical environment within its host may have causal influences. Report the anatomical entity(s) which are in the direct environment of the symbiotic host organism being sampled and which you believe have significant causal influences on your sample or specimen. For example, if the symbiotic host organism being sampled is an intestinal worm, its local environmental context will be the term for intestine from UBERON (http://uberon.github.io/).	0001325
		host of the symbiotic host environmental medium	O	Report the environmental material(s) immediately surrounding the symbiotic host organism at the time of sampling. This usually will be a tissue or substance type from the host, but may be another material if the symbiont is external to the host. We recommend using classes from the UBERON ontology, but subclasses of ‘environmental material’ (http://purl.obolibrary.org/obo/ENVO_00010483) may also be used. EnvO documentation about how to use the field: https://github.com/EnvironmentOntology/envo/wiki/Using-ENVO-with-MIxS. Terms from other OBO ontologies are permissible as long as they reference mass/volume nouns (e.g., air, water, blood) and not discrete, countable entities (e.g., intestines, heart).	0001326
		host of the symbiotic host taxon id	O	NCBI taxon id of the host of the symbiotic host organism.	0001306
		host of the symbiotic host subject id	O	A unique identifier by which each host of the symbiotic host organism subject can be referred to, de-identified, e.g. #H14.	0001327
Core		Relationship to other samples^a^	C	indicates the direct relationship with another sample from the same Bioproject. Accepted terms are: ‘technical replicate of’, ‘after’, ‘before’, ‘next to’, ‘within’, and ‘contains’. Can be repeated to reveal relationship to many samples.	TBD
		Biotic relationship	C	Description of relationship(s) between the subject organism and other organism(s) it is associated with. E.g., parasite on species X; mutualist with species Y. The target organism is the subject of the relationship, and the other organism(s) is the object.	0000028
Other^b^		Observed host symbionts	O	The taxonomic name of the organism(s) found living in mutualistic, commensalistic, or parasitic symbiosis with the specific host.	0001309

*Req* requirements, *O* optional, *C* conditional, *M* mandatory, *TBD* to be determined.

^a^Term may be slightly amended when included to all packages.

^b^Term added to host-associated, human-associated, plant-associated, human-vaginal, human-skin, human-oral, and human-gut packages.

**Fig. 1:**
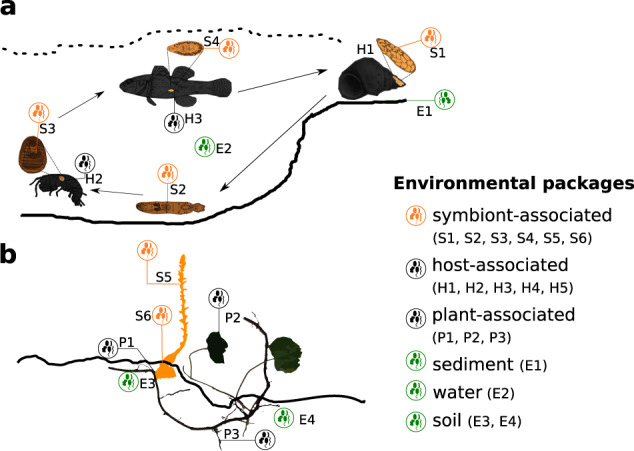
Examples of study design for the sampling of microbes of symbiotic organisms, their hosts and environment. **a** Trematode *Coitocaecum parvum* different life stages (S1, S2, S3, S4) are reported with the MIxS-SA package. The microbiome of infected snail (H1), amphipod (H2) and fish (H3) hosts are reported with the MIxS host-associated package. In addition, the microbiome of environmental sediment (E1) and water (E2) from which these organisms were collected can be reported with MIxS-sediment and MIxS-water, respectively. The following relationships are reported: S1 “within” H1, S2 “within” E2, S3 “within” H2, S4 “within” H3, H1 “next to” E1, H1 “within” E2, H2 “within” E2, H3 “within” E2, E1 “next to” E2. **b** Angiosperm *Orobanche hederae* (S5, S6) parasitizing a host plant (P1, P2, P3) is reported using the included MIxS-SA and MIxS-PA (plant-associated) packages respectively. In addition, the MIxS-soil package is used to report corresponding soil samples. The following relationships are reported: S6 ‘within’ P1, S5 ‘next to’ S6, P1 ‘next to’ P2, P1 ‘next to’ P3, P2 ‘next to’ P3, P1 ‘within’ E3, P3 ‘within’ E4, E3 ‘next to’ E4.

Given the diversity of symbiotic interactions and that the nature and dependence of such interactions can be context-dependent rather than a fixed trait, it was crucial to define terms and provide value syntax that were inclusive for diverse types of symbioses and also across the symbiont life histories and transmission processes. For example, the term “host dependence” (a mandatory item) and “type of symbiosis” (a conditional item) are discrete but complementary items. While “host dependence” aims to provide a general characterization of the known type of host dependence for the symbiotic organism (e.g., facultative), “type of symbiosis” was specifically designed to further characterize the type of biological interaction established between the symbiotic organism and its respective host at the moment the biological sample was taken (e.g., mutualistic). As a result, the MIxS-SA package features mandatory and conditionally mandatory, and optional features that enable flexibility according to the knowledge of the study system at the time of sampling. Two examples of MIxS-SA-compliant metadata are provided in Supplementary information (SI-2), and the respective study designs are presented in Fig. 1. The examples refer to 16 S rRNA gene studies of (a) the bacterial communities of the parasite *Coitocaecum parvum*, a trematode, across four of its life stages: the sporocyst, the metacercaria and the adult, as well as the free-living cercaria [16], and (b) of the leaves and roots of the parasitic plant *Orobanche hederae* and its ivy host, *Hedera* spp. [17].

While identical terms are often used in several of the 17 environmental packages currently available (https://gensc.org/mixs/), here we introduce three additional new terms: one is shared by several relevant MIxS environmental packages, and the two others will feature within the core MIxS package. The new term “observed host symbionts” provides a more comprehensive descriptor for the subject organism associations with smaller symbionts and it has been added to the host-associated, human-associated, plant-associated, human-vaginal, human-skin, human-oral and human-gut packages. The term “biotic relationship” has been added to the core package as a conditional descriptor of the relationship between the subject organism and other larger host organism(s). Finally, it appears necessary to include in the MIxS core a new term that takes into account the nested feature of most associations found in nature, such as host-symbiont-microorganism, in which multiple packages are necessary to describe the samples of the study (e.g., water, sediment, host-associated, and symbiont-associated). The proposed term “relationship to other samples” indicates the direct relationship between two samples from the same Bioproject, that are described in different environmental package(s). This proposed feature, still under development, will allow for integrated analyses of the microbiota of symbiotic organisms and their direct environment, even in the context of co-infections (e.g., symbiont-associated SA1234 is “within” host-associated sample HA8974, “next to” symbiont-associated sample SA7890). This feature will also benefit other studies by providing ecologically-relevant contextual information (e.g., host-associated HA2567 is “within” environmental water sample W1234, “next to” host-associated sample HA5679, ‘next’ to environmental soil sample S5897). In conclusion, it is our hope that the MIxS-SA, together with the new terms, will enable researchers to better conduct integrated analyses of multi-level biological systems with the ultimate goal of better understanding the role of microbes associated with symbionts.

## Supplementary information

Table S1

Table S3
